# A hybrid feature selection algorithm combining information gain and grouping particle swarm optimization for cancer diagnosis

**DOI:** 10.1371/journal.pone.0290332

**Published:** 2024-03-11

**Authors:** Fangyuan Yang, Zhaozhao Xu, Hong Wang, Lisha Sun, Mengjiao Zhai, Juan Zhang

**Affiliations:** 1 Department of Gynecologic Oncology, The First Affiliated Hospital of Henan Polytechnic University, Jiaozuo, Henan, China; 2 School of Computer Science and Technology, Henan Polytechnic University, Jiaozuo, Henan, China; UNITEN: Universiti Tenaga Nasional, MALAYSIA

## Abstract

**Background:**

Cancer diagnosis based on machine learning has become a popular application direction. Support vector machine (SVM), as a classical machine learning algorithm, has been widely used in cancer diagnosis because of its advantages in high-dimensional and small sample data. However, due to the high-dimensional feature space and high feature redundancy of gene expression data, SVM faces the problem of poor classification effect when dealing with such data.

**Methods:**

Based on this, this paper proposes a hybrid feature selection algorithm combining information gain and grouping particle swarm optimization (IG-GPSO). The algorithm firstly calculates the information gain values of the features and ranks them in descending order according to the value. Then, ranked features are grouped according to the information index, so that the features in the group are close, and the features outside the group are sparse. Finally, grouped features are searched using grouping PSO and evaluated according to in-group and out-group.

**Results:**

Experimental results show that the average accuracy (*ACC*) of the SVM on the feature subset selected by the IG-GPSO is 98.50%, which is significantly better than the traditional feature selection algorithm. Compared with KNN, the classification effect of the feature subset selected by the IG-GPSO is still optimal. In addition, the results of multiple comparison tests show that the feature selection effect of the IG-GPSO is significantly better than that of traditional feature selection algorithms.

**Conclusion:**

The feature subset selected by IG-GPSO not only has the best classification effect, but also has the least feature scale (*FS*). More importantly, the IG-GPSO significantly improves the *ACC* of SVM in cancer diagnostic.

## Introduction

With the rapid increase of cancer incidence and mortality, cancer research has attracted more and more attention [1, 2]. As a genetic mutation-like disease, cancer can be diagnosed and treated by analyzing the genes that are mutated at the molecular level [3, 4]. The traditional diagnostic methods usually use cell morphology and histopathology [5, 6]. However, such methods can not reach the clinical requirements of early diagnosis and treatment [7, 8]. Machine learning, as a new technology, is widely used for cancer diagnosis because of its fast operation and high efficiency [9, 10]. Machine learning based cancer diagnosis refers to the process of using machine learning algorithms to model and train gene expression data, and predicting unknown gene expression data according to the trained model [11, 12].

Common machine learning algorithms include support vector machines, decision trees, naive Bayes and ensemble learning [13, 14]. Among them, support vector machine, as a new machine learning algorithm based on statistical theory learning, has been widely used in cancer diagnosis because of its advantages in high-dimensional small sample data. For example, Huang et al. proposed to optimize support vector machine by using fruit fly optimization algorithm, and applied the optimized support vector machine to breast cancer diagnosis [15]. Similarly, Wang et al. [16] proposed to use the improved whale optimization algorithm for feature selection while optimizing support vector machine parameters. Experimental results show that the proposed algorithm is effective in cancer diagnosis.However, due to the large number of cancer-related genes in gene expression data, and the complex interactions and contradictions among these genes [17], SVM is faced with problems such as high time complexity and poor classification effect when processing such data [18, 19].

For high-dimensional feature space and highly feature correlation of gene expression data, feature selection is required before processing these data [20, 21]. Feature selection [22] can simplify the machine learning model, reduce the training time, and improve the diagnostic effect of the model. According to whether it is related to the classification algorithm, it can be divided into filter [23], wrapper [24] and hybrid [25] algorithms. Filter algorithm uses metric to enhance the correlation between features and class, and reduce the correlation between features. Among them, information gain (IG) [26], Chi-square (Chis) [27] and pearson correlation coefficient (pearson) [28] are common metrics in filter algorithms. Unfortunately, the feature subset selected by filter algorithm have poor classification effect and need to set thresholds artificially, which has great blindness [29].

Wrapper [24] algorithm uses search algorithm to search the original features, and takes ACC index of classification algorithm as the metric of selected feature subset. Common search algorithms include particle swarm optimization (PSO) [30], genetic algorithm (GA) [31] and ant colony algorithm (ant colony algorithm). ACA) [32]. However, the time complexity of such algorithms is extremely high [33], and the search algorithm is easy to fall into local optimal solutions during the search process, resulting in that the selected feature subset is not globally optimal [34, 35]. In recent years, some scholars put forward the strategy of combining filter and wrapper. For example, Got et al. [25] proposed a hybrid algorithm combining mutual information and whale optimization algorithm. The features below the threshold are filtered by calculating the mutual information of the features. The filtered features are then searched using the whale optimization algorithm, and the searched subset of features is evaluated based on *ACC*. Similarly, Liu et al. [36] proposed a hybrid algorithm combining gain ratio and multi-objective genetic algorithm. Invalid features are filtered by calculating the gain ratio of features. Then multi-objective genetic algorithm is used to search the filtered features, and the selected feature subset is evaluated according to the *ACC* and the feature scale (*FS*). However, the hybrid algorithm does not solve the problem of blindness in threshold setting and the tendency of search algorithm to fall into local optimal solution [37].

Based on the above description, this paper proposes a hybrid algorithm combining information gain and grouped particle swarm (IG-GPSO). The algorithm first computs the information gain value of each feature and sorts it in descending order according to the value size. Then the sorted features are grouped according to the information gain index, and the features with similar information gain values are divided into a group. Finally, the grouped features are searched using the grouped particle swarm optimization algorithm, and the selected feature subset are evaluated according to two methods: in-group and out-group. The experiment is divided into three parts. The firstly is the feature selection process experiment to observe the working principle of the IG-GPSO algorithm. Then, a comparison experiment with other feature selection algorithms is conducted to verify the effectiveness of the IG-GPSO algorithm. Finally, a comparison experiment with other classification algorithms is done to verify the applicability of the IG-GPSO algorithm. In addition, we also select statistical experiments to verify whether the IG-GPSO algorithm is significantly different from other feature selection algorithms.

1. For the blind threshold setting of filter, this paper proposes a feature ranking strategy. The IG value of features is calculated and ranked according to the size of the value. Unlike traditional filter algorithms, at this stage we only rank the features and do not filter any features.
2. For the high time complexity of wrapper, this paper proposes a feature grouping strategy. By calculating the number of groups and grouping the ranked features according to the information index strategy, the number of features in each group is significantly less than the original number of features.
3. For the PSO is easy to fall into local optimal solutions, this paper proposes a group search strategy. In the in-group evaluation, we use *ACC* as a fitness function. In the out-group evaluation, we use *ACC* and *FS* as fitness function.
4. Experimental results show that the *ACC* and number of *FS* selected by the IG-GPSO algorithm are significantly better than the traditional feature selection algorithm. In addition, statistical experiments show that the IG-GPSO algorithm is significantly different from the traditional feature selection algorithm.

The rest of this paper is organized as: the Datasets and methods section presents the gene expression datasets and the IG-GPSO. The Experimental result section presents the comparative and statistical experiments. The Experiment discussion and analysis section is dedicated to the experimental discussion and analysis, and the Conclusion and future work section is the conclusion and future work.

## Datasets and methods

### Gene expression datasets

In this paper, we selected 4 publicly available gene expression datasets [38, 39], namely Prostate-GE, TOX-171, GLIOMA and Lung-discrete. Prostate-GE contains 5966 genes and 102 samples, including a total of 2 classes of information. TOX-171 contains 5748 genes and 171 samples, including a total of 4 classes of information. GLIOMA contains 4434 genes and 50 samples, including a total of 4 classes of information. Lung-discrete contains 325 genes, 73 samples, and a total of 4 classes of information. All in all, these gene expression data are characterized by a large number of features and a small number of samples.

### Support vector machine

SVM map low-dimensional samples into high-dimensional Spaces and obtain a hyperplane to maximize the margin between the two types of samples [40]. This strategy effectively avoids the phenomenon of “overfitting”, especially when classifying high-dimensional small sample data [41]. Suppose that the training sample set is a binary classification problem, based on statistical theory, the classification model of SVM can be constructed as follows:

$$\begin{array}{c} \left\{ \begin{array}{c} \text{min}\frac{1}{2}(w^{T}w)+C\sum_{i=1}^{l}\xi_{i}\\ s.ty_{i}((w^{T}x_{i})+b)\geq 1-\xi_{i},i=1,2,\cdots ,l \end{array}\right. \end{array}$$ (1)

Where, *C* > 0 is the regularization parameter, *ξ*_*i*_ is the slack variable, *w* ∈ *n* is the normal vector of the classification hyperplane, and *b* is the threshold. Using the KKT [42] condition and duality theory in optimization theory, the optimized model of the dual function can be obtained as follows:

$$\begin{array}{c} \left\{ \begin{array}{c} \text{max}\sum_{i=1}^{l}\alpha_{i}-\frac{1}{2}\sum_{i=1}^{l}\sum_{j=1}^{l}\alpha_{i}\alpha_{j}y_{i}y_{j}(x_{i}^{T}x_{j})\\ s.t\sum_{i=1}^{l}y_{i}\alpha_{i}=0\\ 0\leq \alpha_{i}\leq C;i=1,2,\cdots ,l;j=1,2,\cdots ,l \end{array}\right. \end{array}$$ (2)

Where, *α*_*i*_ is the Lagrange multiplier. The optimization model is a convex quadratic programming problem, so the local optimal solution is the global optimal solution. If $\alpha^{*}=(\alpha_{1}^{*},\alpha_{2}^{*},\cdots ,\alpha_{l}^{*}{)}^{T}$ is the global optimal solution of the model, then:

$$\begin{array}{c} w^{*}=\sum_{i=1}^{l}y_{i}\alpha_{i}^{*}x_{i} \end{array}$$ (3)

According to the KKT complementarity condition given in optimization theory, the optimal solution must satisfy:

$$\begin{array}{c} \alpha_{i}((y_{i}(w^{T}x_{i})+b)-1+\xi_{i})=0,i=1,2,\cdots ,l \end{array}$$ (4)

$$\begin{array}{c} (C-\alpha_{i})\xi_{i}=0,i=1,2,\cdots ,l \end{array}$$ (5)

According to Eqs (3), (4) and (5), the samples corresponding to the Lagrange multiplier lose their effect on the classification problem, while only the samples corresponding to the Lagrange multiplier *α*_*i*_ > 0 play a role, thus deciding the result of the classification.

Support vectors are usually only a small subset of the total sample. After solving the above problem [43], the optimal linear classifier can be obtained as follows:

$$\begin{array}{c} f(x)=\text{sgn}\{(w^{*T}x)+b^{*}\}=\text{sgn}\{\sum_{i=1}^{l}\alpha_{i}^{*}y_{i}(x_{i}^{T}x)+b^{*}\} \end{array}$$ (6)

Where, sgn() is the sign function, and b* is the threshold for classification, which can be obtained from any support vector.

For the nonlinear separable case, SVM constructs the optimal classification in the higher dimensional feature space by mapping the input vector to the higher dimensional feature space. Applying the transformation Φ of *x* from the input space *R*^*n*^ to the feature space *H*, get:

$$\begin{array}{c} x\to \Phi (x)=(\Phi_{1}(x),\Phi_{2}(x),\cdots ,\Phi_{l}(x){)}^{T} \end{array}$$ (7)

Replacing the input vector *x* with the eigenvector Φ(*x*) gives the optimal classification:

$$\begin{array}{c} f(x)=\text{sgn}\{(w^{*T}\Phi (x))+b^{*}\}=\text{sgn}\{\sum_{i=1}^{l}\alpha_{i}^{*}y_{i}(\Phi_{i}{\left(x\right)}^{T}\Phi (x))+b^{*}\} \end{array}$$ (8)

In the above duality problem, the objective function and the classification function are only concerned with the inner product calculation of the training samples. This strategy effectively avoids the complicated calculation of high dimensional space and only needs to calculate the inner product [43].

### Methodology

**Fig 1:**
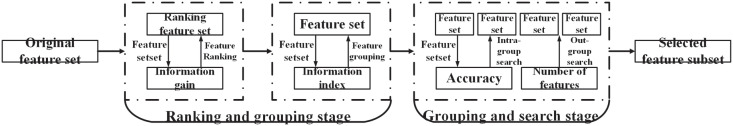
The flowchart of the IG-GPSO.

High-dimensional gene expression data greatly affect the diagnostic *ACC* of machine learning algorithms. Therefore, a hybrid feature selection algorithm combining information gain and grouping particle swarm optimization is proposed in this paper. The first is the ranking and grouping stage, which uses IG to rank the features and groups them according to the information index. Then there is the grouping and search stage, which uses the group PSO to search the features after the group, and evaluates them according to the out–group and in-group. Fig 1 shows the flowchart of the IG-GPSO.

#### A information gain-based ranking and grouping algorithm

Filter algorithm based on information gain calculates the IG value of features, and selects the features whose IG value is higher than the threshold as the selected feature subset [44]. The larger the IG value of the feature, the greater the amount of information contained in the feature. In general, the higher the IG value of a feature, the more distinguishing ability the feature has. Then, the IG of feature *f* is defined as follows:

$$\begin{array}{c} IG(f)=H(f)-H(f|S) \end{array}$$ (9)

In Eq (9), *H*(*f*) is the information entropy of the feature. The larger the value of *H*(*f*), the more information it carries. Then, the information entropy [45] of feature *f* is defined as follows:

$$\begin{array}{c} H(f)=-\sum_{i=1}^{m}P(C_{i}){\text{log}}_{2}P(C_{i}) \end{array}$$ (10)

Where, *P*(*C*_*i*_) is the probability that any sample belongs to class *C*. *P*(*C*_*i*_) = *S*_*j*_/*S*, *m* is the number of sample classes. *S*_*j*_ is the number of samples belonging to class *C*_*i*_ and *S* is the total number of samples.

In Eq (9), *H*(*f*|*S*) is the conditional entropy of feature *f*, which represents the amount of information that feature *f* contains given *S*. Then, the conditional entropy of feature *f* is defined as follows:

$$\begin{array}{c} H(f|S)=-\sum_{i=1}^{m}\sum_{j=1}^{n}P(C_{i},S_{j}){\text{log}}_{2}P(C_{i}|S_{j}) \end{array}$$ (11)

*H*(*f*, *S*) is the joint entropy of sample *S* and feature *f*, which represents the amount of information contained in feature *f* given *S* and *f*. Then, the joint entropy of sample *S* and feature *f* is defined as follows:

$$\begin{array}{c} H(f,S)=-\sum_{i=1}^{m}\sum_{j=1}^{n}P(C_{i},S_{j}){\text{log}}_{2}P(C_{i},S_{j}) \end{array}$$ (12)

In Eq (12), *P*(*C*_*i*_, *S*_*j*_) = *S*_*ij*_/*S*_*j*_ is the probability that the sample in *S*_*j*_ belongs to class *C*_*i*_. in Eq (9) is the IG of this partition obtained from *f*, which represents that the feature with the highest IG is the discriminative feature in a given record set.

Filter algorithm selects the features whose IG value is higher than the threshold as the selected feature subset. However, the artificial setting of the threshold has great blindness. Therefore, this paper proposes a information gain-based ranking and grouping algorithm. First, we give the required number of groups:

$$\begin{array}{c} k=\sqrt[{\gamma }]{\sum_{i=1}^{M}|f_{i}|} \end{array}$$ (13)

Where, *γ* is the feature index of the dataset. || is the *FS* of the statistics. The number of features in each group and the sum of the IG can be calculated by the number of groups. Then, the information index of each grouping can be defined as:

$$\begin{array}{c} I\_index=\sum_{i=1}^{M}|IG(f_{i})|/k \end{array}$$ (14)

The purpose of feature grouping is to combine the features with close IG values, so that the IG values of the features in the group are close, and the IG values of the features outside the group is sparse. Then grouped features are re-ranked according to the IG value. **Algorithm 1** presents the steps of the IG.

**Algorithm 1**: A information gain-based ranking and grouping algorithm (IG)

**Require**: Feature set: *F* = {*f*_1_, *f*_2_, ⋯, *f*_*m*_}; Number of feature groups: *k*

**Ensure**: Feature groups: *F* = {*F*_1_, *F*_2_, ⋯, *F*_*k*_}

  **for** *i* = 1, 2, ⋯, *m* **do**

 1: Calculate the information entropy, conditional entropy and joint entropy of feature *f* according to Eqs (10), (11) and (12).

 2: Calculate the IG value of the feature according to Eq (9).

 3: Ranked feature set: *IG*(*F*) = {*IG*(*f*_1_), *IG*(*f*_2_), ⋯, *IG*(*f*_*m*_)}

  **for** *i* = 1, 2, ⋯, *m* **do**

 4: Calculate the number *k* of required groups according to Eq (13).

 5: Calculate the information index of each group according to Eq (14).

 6: The ranked feature sets are grouped: *F* = {*F*_1_, *F*_2_, ⋯, *F*_*k*_}

  **return** Feature groups: *F* = {*F*_1_, *F*_2_, ⋯, *F*_*k*_}

#### A grouping particle swarm optimization-based wrapper algorithm

*Particle parameter setting. PSO is a meta-heuristic search algorithm that simulates the foraging behavior of birds. In the search process, the swarm treats particles as points in space, and these particles search at a certain speed, adjusting their flight speed and direction based on their own flight experience and the flight experience of other particles.

Suppose the *i*-th particle has position *X*_*i*_ = (*x*_*i*1_, *x*_*i*2_, ⋯, *x*_*iN*_) and velocity *V*_*i*_ = (*v*_*i*1_, *v*_*i*2_, ⋯, *v*_*iN*_) [46]. The best position through which particle passes is denoted as *P*_*i*_ = (*p*_*i*1_, *p*_*i*2_, ⋯, *p*_*iN*_). The best position that all particles passes through is denoted as *P*_*gbest*_ = (*p*_*g*1_, *p*_*g*2_, ⋯, *p*_*gN*_). Then, update the position and velocity of each particle according to Eqs (15) and (16) in the iterative search:

$$\begin{array}{c} v_{in}^{k+1}=w\times v_{in}^{k}+c_{1}r_{1}(p_{in}^{k}-x_{in}^{k})+c_{2}r_{2}(p_{gn}^{k}-x_{in}^{k}) \end{array}$$ (15)

$$\begin{array}{c} x_{id}^{k+1}=x_{id}^{k}+v_{id}^{k+1} \end{array}$$ (16)

Where, *w* is the inertia weight; *k* is the number of iterations; *r*_1_ and *r*_2_ are random numbers between 0 and 1. *c*_1_ and *c*_2_ are used to accelerate the particle. Moreover, *v*_*in*_ is limited by the maximum velocity *v*_max_, and if the velocity of a particle in a dimension exceeds the maximum velocity *v*_max_, then the velocity of the particle in that dimension is limited below the maximum velocity [47].

In the search process, *c*_1_ is used to control the convergence speed of the particle, and *c*_2_ is used to control the search speed of the particle. When *c*_2_ = 0, the velocity of the particle will no longer change, resulting in the failure to find the optimal position, and then fall into the local optimal solution [48]. Based on this, this paper proposes a grouping particle swarm optimization-based wrapper algorithm.

In this algorithm, the speed and position of the particles are updated by adding the previously grouped feature groups to the search space when the particles fall into the local optimal solution. To this end, we also propose in-group and out-group particle fitness functions.

*Particle initialization. Grouping PSO-based Wrapper algorithm refers to the process of selecting *M* optimal features from *N* features (*M* ≤ *N*). Each particle in a particle swarm during the search process represents a potentially optimal subset of features. For each particle, if the *i*-th bit is 1, it means the feature is selected, otherwise it means the feature is not selected. Thus, each particle represents a potentially optimal subset of a subset of features.

In the feature search process, PSO is easy to be affected by a single particle, and then fall into the local optimal solution, so it can not find the global optimal solution. Therefore, we initialize the feature set using a grouping strategy. Suppose that the number of features corresponding to each particle is *P*. In each search round, *P* is *N* of the total number of features. When the PSO falls into the local optimal solution, the diversity of particles is improved by adding a new feature group.

*Fitness function. Wrapper algorithm based on PSO generally use the *ACC* of the classification algorithm as the fitness function to evaluate the feature subset. Instead, this paper proposes two fitness functions, in-group and out-group, to evaluate the selected feature subset. In in-group evaluation, we use *ACC* as a fitness function for the selected feature subset. Specifically defined as follows:

$$\begin{array}{c} F\text{int}ness(F_{j})=ACC(F_{j}) \end{array}$$ (17)

Where, *ACC* is the performance index of the classification algorithm. In the out-group evaluation, we use the *ACC* and the *FS* as the fitness function for the selected feature subset. Specifically defined as follows:

$$\begin{array}{c} F\text{int}ness(F_{j})=\lambda \times ACC(F_{j})-(1-\lambda )\times \frac{Size(F_{j})}{Size(F)} \end{array}$$ (18)

Where, *Size*() is used to count the *FS*. λ is the weight of the *FS*, which is used to balance the *ACC* and the *FS*. According to Eq (18), the feature subset selected based on the out-group fitness function has the least *FS* and the best *ACC*.

*Algorithm description and analysis. For the problem that PSO is easy to fall into local optimal solution, this paper proposes a grouping particle swarm optimization-based wrapper algorithm. The algorithm searches for grouped features and evaluates them using in-group and out-group fitness functions. In the in-group evaluation, we use *ACC* as the evaluation function. In out-group evaluation, we use *ACC* and *FS* as fitness functions.

Step 1 is the feature input process, which is used to sequentially output the feature group *F*_*j*_. Step 2 is the particle initialization process, which is used to initialize the feature group *F*_*j*_. Steps 3-6 are the in-group feature evaluation process, which is used to search and evaluate the feature *F*_*j*_, and output the optimal feature group $F_{j}^{\prime }$ in this round. Steps 7-8 are the out-group feature evaluation process, which is used to evaluate the searched $F_{j}^{\prime }$ and output the globally optimal feature group.

**Algorithm 2**: A grouping particle swarm optimization-based Wrapper algorithm (GPSO)

**Require**: Feature groups: *F* = {*F*_1_, *F*_2_, ⋯, *F*_*k*_}

**Ensure**: Optimal feature groups: $F^{\prime }=\{F_{1}^{\prime },F_{2}^{\prime },\cdots ,F_{k}^{\prime }\}$

  **for** *j* = 1, 2, ⋯, *k* **do**

 1: Input the corresponding feature groups in turn.

  **for** *i* = 1, 2, ⋯, *Size*(*F*_*j*_) **do**

   2: Initialize the position and velocity of each particle *i*, and set the current optimal particle as *pbest*_*i*_ and *gbest*_*i*_.

  **for** *i* = 1, 2, ⋯, *Size*(*F*_*j*_) **do**

   3: Calculate the fitness of each particle *i* in the group according to Eq (17).

   4: For each particle *i*, compare *F*i*tness*_*i*_ with the local size *pbest*_*i*_. If *F*i*tness*_*i*_ is better than *pbest*_*i*_, then *pbest*_*i*_ = *F*i*tness*_*i*_.

   5: For each particle *i*, compare *F*i*tness*_*i*_ with the global size *gbest*_*i*_. If *F*i*tness*_*i*_ is better than *gbest*_*i*_, then *gbest*_*i*_ = *F*i*tness*_*i*_.

   6: Update the position and velocity of particle *i* according to Eqs (15) and (16).

 7: Calculate the fitness of the feature group outside the group according to Eq (18).

 8: For feature groups *F*_*j*_, compare *F*i*tness*_*i*_ with the size of the global *F*i*tness*_*j*_. If *F*i*tness*_*i*_ is better than *F*i*tness*_*j*_, then *F*i*tness*_*j*_ = *F*i*tness*_*i*_

   **return** Optimal feature groups: $F^{\prime }=\{F_{1}^{\prime },F_{2}^{\prime },\cdots ,F_{k}^{\prime }\}$

## Experimental result

### Experimental setting

Feature selection algorithms usually use the classification index of classification algorithms to evaluate the goodness of the selected feature subset. In addition, we also select the *FS* as an evaluation index for the algorithm. In this section, 11 feature selection algorithms are selected for comparison experiments. Among them, the information gain (IG) [26], chi-square (Chis) [27] and pearson correlation coefficient (Pearson) [28] are filter algorithms. Particle swarm optimization (PSO) [30], genetic algorithm (GA) [31] and ant colony algorithm (ACA) [32] are wrapper algorithms. The combination of information gain and particle swarm optimization (IG-PSO) [26, 30], information gain and genetic algorithm (IG-GA) [26, 31], information gain and ant colony algorithm (IG-ACA) [26, 32], chi-square and particle swarm optimization (Chis-PSO) [27, 30], and pearson correlation coefficient and particle swarm optimization (Pearson-PSO) [28, 30] are hybrid algorithms.

The comparison experiment is divided into three parts: firstly, the experiments on the feature selection process, in which the feature ranking, feature grouping and feature selection process of the IG-GPSO is observed by setting the interrupt procedure. Then the comparison experiment with other algorithms is carried out, and the IG-GPSO is compared with 11 traditional feature selection algorithms to verify the effectiveness. Finally, comparison experiment with other classification algorithms, SVM is compared with KNN to verify the applicability. In addition, the multiple comparison tests between the IG-GPSO and 11 traditional feature selection algorithms is performed to verify whether the IG-GPSO and 11 traditional feature selection algorithms have significant differences.

### Experiments on the feature selection process

**Fig 2:**
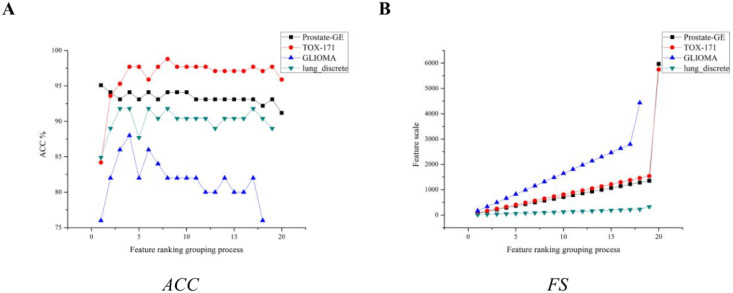
The feature ranking grouping process of the IG-GPSO. (A)Accuracy of the feature ranking grouping process. (B)The number of feature subsets of the feature ranking grouping process.

This section presents experiments on the feature selection process, in order to observe the influence of the threshold on the IG-GPSO, we use the IG to rank the features, and the ranked features were grouped according to the information index. The threshold of the filter stage is set to 1, 2, ⋯, *k*. In addition, we use SVM to test the selected feature subset. Fig 2 shows the feature ranking grouping process of the IG-GPSO.

**Fig 3:**
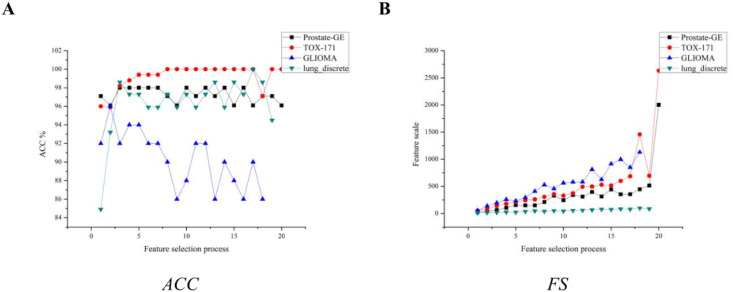
The feature selection process of the IG-GPSO. (A)Accuracy of the feature selection process. (B)The number of feature subsets of the feature selection process.

Observing Fig 2, the feature selection results of IG on different datasets show different trends, respectively. On the lung-discrete dataset, when the threshold is 2, the *ACC* index of SVM reaches 91.8%, and the overall trend is increasing. On the Prostate-GE dataset, with the increase of the threshold, the classification effect of SVM shows a downward trend, and the *ACC* index reaches 91.2% when the threshold is 20. On the TOX-171 dataset, with the increase of the threshold, the SVM reaches the maximum value when the threshold is 8, and the *ACC* index is 98.8%. In summary, the threshold may vary greatly depending on the dataset. Fig 3 shows the feature selection process of the IG-GPSO.

Observing Fig 3, the classification effects of SVM on different datasets also show different trends. At the beginning of the out-group search, the classification effect of SVM is relatively poor, especially on the TOX-171 dataset, where the *ACC* index is only 96.0%. This is mainly because the number of features in the group is very small, which makes the PSO fall into a local optimal solution when searching. With the increase of out-group search, except for GLIOMA dataset, the classification effect of SVM showed an increasing trend. On lung-discrete and TOX-171 datasets, the *ACC* index of SVM achieves 100.0%. This result shows that the group search strategy effectively avoids the situation of the PSO falling into the local optimal solution.

### Comparison experiments with other feature selection algorithms

This section presents the comparison experiments with other feature selection algorithms, and we select 11 traditional feature selection algorithms for experiments. Among them, the threshold of the filter algorithm is set to half of the total number of informative features. The evaluation algorithm of the wrapper algorithm is the SVM. The participation of the hybrid algorithm is the same as those for the filter and wrapper algorithms. Tables 1 and 2 shows the *ACC* and *FS* results of SVM on the selected feature subset.

**Table 1:** *ACC* index of SVM on the selected feature subset.

Dataset	ALL	IG	Chis	Pearson	PSO	GA	ACA	IG-PSO	IG-GA	IG-ACA	Chis-PSO	Pearson-PSO	IG-GPSO
*Prostate-GE*	91.2	94.1	93.1	91.2	96.1	92.2	93.1	**98.0**	97.1	**98.0**	**98.0**	95.1	**98.0**
*TOX-171*	95.9	97.7	97.7	98.8	99.4	**100.0**	98.8	**100.0**	98.8	**100.0**	**100.0**	**100.0**	**100.0**
*GLIOMA*	76.0	82.0	82.0	82.0	84.0	84.0	82.0	90.0	88.0	90.0	95.0	84.0	**96.0**
*Lung-discrete*	89.0	89.0	91.8	89.0	94.5	93.2	95.9	97.3	94.5	98.6	94.5	94.5	**100.0**
*Average*	88.03	90.70	91.15	90.25	93.50	92.35	92.45	96.33	94.60	96.65	96.88	93.40	**98.50**

**Table 2:** *FS* index of SVM on the selected feature subset.

Dataset	ALL	IG	Chis	Pearson	PSO	GA	ACA	IG-PSO	IG-GA	IG-ACA	Chis-PSO	Pearson-PSO	IG-GPSO
*Prostate-GE*	5966	677	677	2983	2525	2927	2380	283	331	285	130	1141	**63**
*TOX-171*	5748	769	769	2874	1732	2325	2314	338	388	364	363	1198	**305**
*GLIOMA*	4434	1398	1398	2217	1561	1856	2010	426	590	530	317	1076	**138**
*Lung-discrete*	325	111	111	163	142	153	139	52	**48**	53	50	59	77
*Average*	4118.25	738.75	738.75	2059.25	1490.00	1815.25	1710.75	274.75	339.25	308.00	215.00	869.50	**145.75**

From Tables 1 and 2, the average *ACC* index of SVM on the original gene expression datasets is only 88.03%, especially the *ACC* index on the GLIOMA dataset is only 76.0%. This shows that the high-dimensional feature space and high feature redundancy of data greatly damage the classification effect of SVM. Compared with the filter algorithm, IG has the best feature selection effect on the Prostate-GE dataset, while Chis has the best feature selection effect on the Lung-discrete dataset. Overall, Chis has the best feature selection effect in the filter algorithms, and the average *ACC* index of SVM is 91.15%. Compared with the wrapper algorithm, PSO has the best feature selection effect, and the average *ACC* index of SVM is 93.50%.

Compared with the hybrid algorithm, Chis-PSO has the best feature selection effect on the TOX-171 dataset, and the *ACC* index of SVM is 98.0%, and the *FS* index is 130. IG-ACA has the best feature selection effect on the Lung-discrete dataset, the *ACC* index of SVM is 98.6%, and the *FS* index is 53. Overall, Chis-PSO has the best feature selection effect in the hybrid algorithms. Moreover, the feature selection effect of the hybrid algorithm is significantly better than the filter and wrapper algorithms. Compared with the traditional feature selection algorithms, the feature selection effects of the IG-GPSO are optimal, and the average *ACC* index of the SVM is 98.50%. In addition, the *FS* index of the IG-GPSO is also the least. This shows that the IG-GPSO effectively avoids the blindness of threshold setting and the PSO is easy to fall into a local optimal solution.

### Comparison experiments with other classification algorithms

This section presents the comparison experiments with other classification algorithms, in order to avoid the limitations brought by using a single evaluation algorithm, we select KNN as the evaluation algorithm for the wrapper and hybrid algorithms. Similarly, we also selected 11 traditional feature selection algorithms for comparative experiments, and used KNN to test the datasets after feature selection. Tables 3 and 4 shows the *ACC* and *FS* results of KNN on the selected feature subset.

**Table 3:** *ACC* index of KNN on the selected feature subset.

Dataset	ALL	IG	Chis	Pearson	PSO	GA	ACA	IG-PSO	IG-GA	IG-ACA	Chis-PSO	Pearson-PSO	IG-GPSO
*Prostate-GE*	85.3	91.2	90.2	87.3	90.2	88.2	91.2	94.1	92.2	95.1	94.1	91.2	**96.1**
*TOX-171*	85.4	89.5	90.6	89.5	95.3	91.2	93.6	97.1	94.7	95.9	**97.7**	95.9	**97.7**
*GLIOMA*	72.0	82.0	82.0	78.0	86.0	76.0	84.0	82.0	90.0	92.0	86.0	86.0	**94.0**
*Lung-discrete*	83.6	86.3	84.9	83.6	93.2	90.4	91.8	94.5	94.5	**97.3**	**97.3**	94.5	**97.3**
*Average*	81.58	87.25	86.93	84.60	91.18	86.45	90.15	91.93	92.85	95.08	93.78	91.90	**96.28**

**Table 4:** *FS* index of KNN on the selected feature subset.

Dataset	ALL	IG	Chis	Pearson	PSO	GA	ACA	IG-PSO	IG-GA	IG-ACA	Chis-PSO	Pearson-PSO	IG-GPSO
*Prostate-GE*	5966	677	677	2983	2630	2506	2685	269	299	267	224	1283	**27**
*TOX-171*	5748	769	769	2874	1800	2144	1890	287	376	336	**282**	1038	305
*GLIOMA*	4434	1398	1398	2217	1305	968	1319	1398	486	480	726	982	**60**
*Lung-discrete*	325	111	111	163	112	113	126	59	52	49	57	61	**27**
*Average*	4118.25	738.75	738.75	2059.25	1461.25	1432.75	1505.00	503.25	303.25	283.00	322.25	841.00	**104.75**

From Tables 3 and 4, KNN has extremely poor classification effect on the original gene expression datasets, and the average *ACC* index is 81.58%. This shows again that the high-dimensional feature space and high feature redundancy of the data greatly damage the classification effect. Similarly, the average *ACC* index of KNN on the feature subset selected by IG is 87.25%, and the *FS* index is 738.75. PSO has the best feature selection effect in the wrapper algorithms, and the average *ACC* index of KNN is 91.18%. Overall, the *FS* selected by the filter algorithm is less than the wrapper algorithm, but the classification effect of the feature subset selected by the filter algorithm is worse than the wrapper algorithm.

Compared with the hybrid algorithm, IG-ACA has the best feature selection effect on the Prostate-GE dataset, and the *ACC* index of KNN is 95.1%. The Chis-PSO has the best feature selection effect on the TOX-171 dataset, and the *ACC* index of KNN is 97.7%. Overall, IG-ACA has the best feature selection effect in the hybrid algorithms, and the average *ACC* index of KNN is 95.08%. The filter algorithm has the worst feature selection effect, while the wrapper algorithm has the largest *FS* selected. However, the feature selection effect of the hybrid algorithm is significantly better than the filter and wrapper algorithms. More importantly, KNN has the best classification effect on the dataset after the IG-GPSO feature selection, the average *ACC* index is 96.28%, and the *FS* index is also the smallest.

### Statistical experiments with other feature selection algorithms

This section presents statistical experiments, in order to compare whether there are significant differences algorithms, we choose Friedman test for statistical experiments. The ranking values of all algorithms on each dataset are counted. For all algorithms, all ranking values are obtained as comparison values. According to the Friedman test, the following results are obtained:

$$\begin{array}{c} \chi_{F}^{2}=\frac{12\times N}{k\times (k+1)}[\sum_{j=1}^{k}R_{j}^{2}-\frac{k\times {\left(k+1\right)}^{2}}{4}] \end{array}$$ (19)

Where, *N* is the number of datasets, *k* is the number of feature selection algorithms, and *R*_*j*_ is the average of the ranking values of each feature selection algorithm. For computational convenience, we transform the $\chi_{F}^{2}$ distribution into a distribution obeying *F*_*F*_. Specifically:

$$\begin{array}{c} F_{F}=\frac{(N-1)\times \chi_{F}^{2}}{N\times (k-1)-\chi_{F}^{2}} \end{array}$$ (20)

Where, the *F*_*F*_ distribution has *k* − 1 and (*N* − 1) × (*k* − 1) degrees of freedom. Then, the *ACC* and *FS* index results of SVM and KNN on 4 datasets are tested. When the significance level *α* = 0.05, the null hypothesis is that there is no significant difference between all algorithms. According to Eqs (19) and (20), when *N* = 8, the Friedman test result is as follows:

When the significance level *α* = 0.05, *F*(12, 84) = 1.869. The results based on the SVM indexes are: $\chi_{F}^{2}=75.43$, *F*_*F*_ = 25.67. However, *F*_*F*_ is significantly greater than 1.869, which rejects the null hypothesis. Similarly, the results based on the KNN indexes are: $\chi_{F}^{2}=80.19$, *F*_*F*_ = 35.95. However, *F*_*F*_ is significantly greater than 1.869, which again rejects the null hypothesis. In summary, the IG-GPSO is significantly different from traditional feature selection algorithms.

## Experiment discussion and analysis

In experiments on feature selection process, with the increase of threshold, the filter stage of the IG-GPSO shows different trends on different datasets, such as an increasing trend on GLIOMA dataset and a decreasing trend on Prostate-GE dataset. These results show that different thresholds may have different effects for different datasets. Therefore, there is a great blindness in setting the threshold artificially. The filter and wrapper stage of the IG-GPSO also shows different trends on different datasets. Overall, with the increase of out-group search, the *ACC* index of SVM on all datasets shows an upward trend. These results show that the feature grouping stage of the IG-GPSO reduces the time complexity of the search algorithm and effectively avoids the situation that the PSO is easy to solve locally. Therefore, the IG-GPSO takes into account both time complexity and *ACC*.

Comparison experiments with other feature selection algorithms, in the filter algorithms, Chis has the best feature selection effect, and the average *ACC* index of SVM is 91.15%. Unfortunately, Pearson has the worst feature selection effect, and the average *ACC* index of SVM is 90.25%. In the wrapper algorithms, PSO has the best feature selection effect, and the average *ACC* index of SVM is 93.50%. Similarly, GA has the worst feature selection effect, and the average *ACC* index of SVM is 92.35%. In the hybrid algorithms, Chis-PSO has the best feature selection effect, and the average *ACC* index of SVM is 96.88%. Pearson-PSO has the worst feature selection effect, and the average *ACC* index of SVM is 93.40%. The difference is that hybrid algorithm has a better feature selection effect than the filter and wrapper algorithm. Therefore, the filter algorithm only reduces the feature space of the data, but does not solve the problem of high feature redundancy.

In addition, the *FS* index by the filter algorithm is a fixed number, and it is half of the total number of informative features. In the wrapper algorithm, the *FS* index by PSO is the least, and the average *FS* index of SVM is 1490.00. The *FS* index by GA is the largest, and the average *FS* index of SVM is 1815.25. Overall, the *FS* index by the wrapper algorithm is not fixed, and it is more than the filter algorithm. The wrapper algorithm effectively avoids the blindness of threshold setting by evaluating the feature subset. The wrapper algorithm effectively avoids the blindness of threshold setting by evaluating the feature subset. However, the time complexity of the search algorithm is very high, which significantly limits the efficiency of the wrapper. In the hybrid algorithm, the *FS* index by Chis-PSO is the least, and the average *FS* index of SVM is 215.00. The *FS* index by Pearson-PSO is the largest, and the average *FS* index of SVM is 869.50. In general, the *FS* index by the hybrid algorithm is much less than the filter and wrapper algorithms.

Comparison experiments with other classification algorithms, in the filter algorithms, KNN still has poor classification effect on the original gene expression datasets, and the average *ACC* index is 81.58%. IG has the best feature selection effect, and the average *ACC* index of KNN is 87.25%. In the wrapper algorithms, PSO has the best feature selection effect, and the average *ACC* index of KNN is 91.18%. The difference is that IG-ACA has the best feature selection effect in the hybrid algorithm, and the average *ACC* index of KNN is 95.08%. Pearson-PSO has the worst feature selection effect, and the average *ACC* index of SVM is 91.90%. In addition, the *FS* index by the hybrid algorithm is less than that of the filter and the wrapper algorithm. Compared with the traditional feature selection algorithm, the feature subset selected by the IG-GPSO has the best classification effect, and the average *ACC* index of KNN is 96.28%. The result shows that no matter which classification algorithm is used as the evaluation algorithm, the feature selection effect of the IG-GPSO is optimal.

To further verify the effectiveness of the IG-GPSO, we use the Friedman test for multiple comparisons. We selected the *ACC* and *FS* indexes of SVM and KNN on the selected feature subset as data values for testing. In multiple comparisons, the assumption is that there is no significant difference between all algorithms. When the significance level *α* = 0.05, the multiple comparison tests based on Friedman are all able to reject the null hypothesis. It can be concluded that no matter which classification algorithm is used as the evaluation algorithm, the feature selection effect of the IG-GPSO is significantly better than the traditional feature selection algorithm. The result shows that the difference of evaluation algorithms does not affect the feature selection effect of the IG-GPSO, and the selected feature subset has certain applicability.

In general, the high-dimensional feature space and high feature redundancy of data greatly harm the classification effect of the classification algorithms. SVM and KNN have a significant improvement in each classification index on the datasets after feature selection. Specifically, Pearson has the worest feature selection effect in the single feature selection algorithms. PSO has the best feature selection effect in the single feature selection algorithms. Chis-PSO and IG-ACA have the best feature selection effect in the traditional feature selection algorithms, and the *FS* index is also the least. Pearson-PSO is even worse than some single algorithms in feature selection, which is mainly due to the fact that Pearson removes some important features in the filter stage. Compared with the traditional feature selection algorithm, the *ACC* and the *FS* indexes by the IG-GPSO are optimal. In addition, SVM has the best classification results in all datasets. Therefore, we use SVM as the applied algorithm for cancer diagnosis.

## Conclusion and future work

Machine learning is widely used in cancer diagnosis. However, due to the inherent high-dimensional feature space and high feature redundancy of gene expression data, the application effect of existing machine learning algorithms is poor. Based on this, this paper proposes a hybrid feature selection algorithm combining information gain and grouping particle swarm optimization. Different from the traditional filter algorithm, we use the information gain to calculate the IG value of each feature, and rank the value in descending order. Furthermore, this paper proposes a information gain-based ranking and grouping algorithm. By grouping the features, the IG of the features in the group is close. Finally, we use the grouping PSO algorithm to search for the grouped features and evaluate them according to both in-group and out-group. Experimental results show that the IG-GPSO has the best feature selection effect, and the *ACC* indexes of SVM and KNN on 4 gene expression datasets is 98.50% and 96.28%, respectively. In addition, multiple comparison tests show that the IG-GPSO is significantly better than traditional feature selection algorithms. SVM has the best classification effect, and we selected SVM as the applied algorithm for the cancer diagnosis. However, on some gene expression datasets, the number of feature subset selected by the IG-GPSO is not the smallest. This may be due to the fact that the feature grouping does not consider the correlation between features, which results in too many feature subsets. Therefore, the future work is to consider introducing mutual information into feature groups in order to screen the feature groups with very low correlation.
